# Effects of timed artificial insemination following estrus synchronization in postpartum beef cattle

**Published:** 2012-01-18

**Authors:** A. Malik, H. Wahid, Y. Rosnina, A. Kasim, M. Sabri

**Affiliations:** 1 *Department of Animal Science, Faculty of Agriculture, Islamic Kalimantan University, Banjarmasin, Indonesia*; 2 *Department of Veterinary Clinical Studies, Faculty of Veterinary Medicine, Universiti Putra Malaysia, 43400 UPM Serdang, Selangor Darul Ehsan, Malaysia*; 3 *Department of Animal Science, Faculty of Agriculture. Universiti Putra Malaysia, 43400 UPM Serdang, Selangor Darul Ehsan, Malaysia*; 4 *Department of Veterinary Pathology and Microbiology, Faculty of Veterinary Medicine, Universiti Putra Malaysia, 43400 UPM Serdang, Selangor Darul Ehsan, Malaysia*

**Keywords:** Cows, Estrus synchronization, CIDR, Timed artificial insemination (TAI), Pregnancy rate

## Abstract

The objectives of this study were to evaluate estrus response and pregnancy rates resulting from timed artificial insemination (AI) following estrus synchronization using CIDR in postpartum beef cattle. A total of 100 cows were randomly divided into three groups. Groups 1, 2 and 3 were artificially inseminated at 48-50 h (n=30), 53-55 h (n=30) and 58-60 h (n=40) after CIDR removal, respectively. Estrus synchronization was carried out using a CIDR containing 1.38 mg progesterone. All cows were given 2 mg estradiol benzoate, intramuscularly on the day of CIDR insertion (D 0). The CIDR was removed after 8 days and 125 μg of prostaglandin F_2_α (PGF2α) was injected intramuscularly. One day after CIDR removal all cows were given 1 mg of estradiol benzoate intramuscularly (D 9). Cows were observed visually for estrus after removal of CIDR. Between 30 and 32 days after timed AI, pregnancy was determined using transrectal ultrasonography. The first estrus observation which is approximately 32 h after CIDR removal showed no significant difference (P>0.05) among the three groups. The onset response of estrus after 32 h removal of CIDR was less than 10% in all three groups 6.6% (G1), 6.8% (G2) and 7.3% (G3). Furthermore, percentages of estrus response (D 10) following CIDR removal were 76.6%, 75.0% and 77.5%. The difference between on D 9 and D 10 estrus response were statistically significant (P<0.05). The pregnancy rates were 23.3% (G1), 26.6% (G2) and 37.5% (G3), which were not significant (P>0.05).

## Introduction

One of the strategies for improving pregnancy rates in the modern beef industry is by utilizing a synchronization program. In cattle, estrus synchronization and artificial insemination (AI) can be used to maximize the reproductive potential of cows by incorporating superior genetics into their operations (Leitmana *et al.*, 2009). Various devices have been used including controlled internal drug releasing (CIDR) protocol, which is an intra vaginal progesterone releasing device for estrus synchronization.

It has been widely used in estrus synchronization in beef cattle (Lucy *et al.*, 2001) and in the treatment of reproductive problems (Lammoglia *et al.*, 1998; Day *et al.*, 2000; Lamb *et al.*, 2001; Todoroki *et al.*, 2001). Kim *et al*. (2007) verified that CIDR-based timed artificial insemination (TAI) protocol is an effective technique to increase the pregnancy rate of non- lactating repeat breeders in dairy cows.

The use of estrus synchronization timed AI protocols is beneficial to many farmers since it reduces the time and labor required for estrus detection (Stephens and Rajamahendran, 1998). It also minimizes the frequency of animal handling (Busch *et al.*, 2008; Leitmana *et al.*, 2009). Successful estrus synchronization protocols that facilitate timed AI would possibly increase the implementation of AI in beef cattle (Patterson *et al.*, 2003).

Progress in estrus synchronization to manage estrus cycles in cows that result in expression of high fertility and ovulation will more readily facilitate timed AI (Patterson *et al.*, 2003). Studies by Bremer *et al*. (2004) and Dobbins *et al*. (2006) confirmed that there is a significant increase in pregnancy with timed AI at 66 h compared with 48 or 72 h after prostaglandin F_2_α (PGF2α) injection. Furthermore, Larson *et al*. (2006) reported that the peak estrus response following after synchronization by CIDR protocols occurred 48 to 60 h after removal of CIDR, and injection of prostaglandin.

To date, researchers have yet to determine the most suitable time for fixed time AI after estrus synchronization by CIDR protocol.

Thus, the objectives of this study were to evaluate estrus response and pregnancy rates resulting from timed AI at 48-50 h, 53-55 h and 58-60 h following estrus synchronization using CIDR in postpartum beef cattle.

## Materials and Methods

This study was conducted in two farms in Serdang Malaysia (Lat: 2^0^ 6N and long: 103^0^ 24^1^ 34E) situated about 50 m above sea level, with average ambient temperature of 30°C and relative humidity of 87.5%. A total of 100 Brangus cows were randomly divided into three groups. Groups 1, 2 and 3 consisted of 30, 30 and 40 cows, respectively. All cows had of at least three to five years of age, an average weight of 550 ± 8.45 kg, 50-55 d postpartum, mean lactation at 2 to 3 times.

All cows were healthy with body condition score of 5-6 scale of 1-9 (Houghton *et al.*, 1990) were selected for this experiment. Non-pregnant status in these cows was confirmed based on record and rectal palpation. All cows were raised under a similar grazing system and supplemented with commercial concentrate of palm kernel cake at the rate of 2 kg/head/day.

### Estrus synchronization

**Fig. 1:**
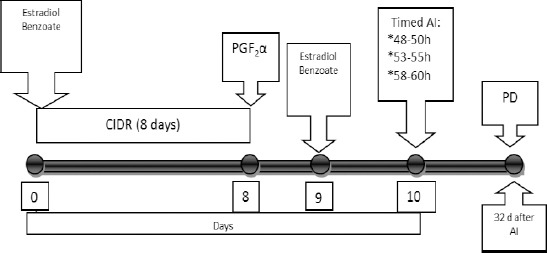
Procedure for estrus synchronization and timed AI in beef cows.

Estrus synchronization was carried out using a CIDR protocol (Pfizer, Animal Health, New Zealand) containing 1.38 mg progesterone. All cows were given 2 mg estradiol benzoate (Cidirol, Biomac Laboratories Ltd) intramuscularly during the day of CIDR insertion (day 0). The CIDR was removed after 8 days and 125 μg PGF2α (Estrumate, Schering-Plough Animal Health, Australia) injected intramuscularly. One day after CIDR removal, all cows were given 1 mg of estradiol benzoate intramuscularly (Figure 1).

### Estrus observation

**Fig. 2:**
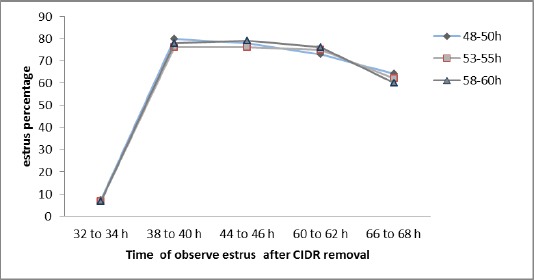
Estrus response was observed after CIDR removal and PGF_2_α injection.


The cows were observed discontinuously in the paddocks for onset, duration and behavioral patterns of estrus every 6 h for 66 h following CIDR removal. All cows were observed visually for mounting, standing to be mounted, and number of mounts performed for a period of two hours, immediately after removal of CIDR and injection of PGF2α (Figure 2). Cows receptive to at least 3 mounts were considered to be in estrus (Acevedo *et al.*, 2007; Busch *et al.*, 2008). The percentage of estrus response was calculated by:

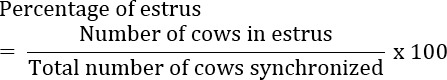


All cows were artificially inseminated using frozen semen base on G1 (48-50 h), G2 (53-55 h) and G3 (58-60 h), after removal of CIDR. Pregnancy diagnosis was conducted 30-32 days after insemination using a 5.0 MHz linear probe attached to an ultrasound scanner (Aloka SSD-500 Echo Camera, Japan).

### Statistical analysis

The proportions of cows that showed estrus after removal of the CIDR and PGF_2_α injection as protocol as well as pregnancy rates were analyzed by separate Chi-square analyses using the Frequency Procedure of SAS Ver. 9.1.3 (SAS, 2006).

## Results and discussions

The first estrus observation which is approximately 32 h (D9) after CIDR removal showed no significant difference (P>0.05) among the three groups. The onset response of estrus after removal of CIDR was less than 10% in all three groups 6.6% (G1), 6.6% (G2) and 7.3% (G3).

Furthermore, percentages of estrus response on D10 (38-60h) following CIDR removal were 76.6%, 75.0% and 77.5%. The difference between on D9 and D10 estrus response were statistically significant (P<0.05). The pregnancy rate was higher in G3 (37.5%) than in G1 (26.6%) and G2 (23.3%) groups (Table 1), but not significantly different (P>0.05).

**Table 1:** Percentages of estrus response and pregnancy rates based on estrus response and total sample after timed AI.

Experiment group (Timed AI)	Total sample	Percentages of estrus response after CIDR removal (38-60h)	Number of pregnant cows	% Pregnancy: based on	
				Estrus response	Total sample
48-50 h	30	23/30 (76.6)	8	9/31 (29.1)	8/30 (26.6)
53-55 h	30	22/30 (75.0)	7	11/30 (36.6)	7/29 (23.3)
58-60 h	40	31/40 (77.5)	15	15/31 (48.4)	15/41 (37.5)

Timing of insemination is very important for successful breeding of cattle in the AI program. One aspect that requires special attention is the estrus synchronization in which it can help to fix the time for AI and thus reduce cost, time and labor required for estrus detection (Bader *et al.*, 2005; Larson *et al.*, 2006; Schafer *et al.*, 2007). There was also no difference observed among the groups throughout observation period.

The percentage of cows in estrus for G1 was higher (77.50%) than 32-34 h after removal of CIDR. This could be due to the peak activity of estradiol which prepare for subsequent ovulation. Similar results were also reported by Zelinski *et al*. (1980) and Busch *et al*. (2008) who suggested that cows that exhibited estrus after removal of CIDR may have attained concentrations of estradiol necessary to effectively prepare follicular cells for luteinisation. Ando *et al*. (2005) reported that every cow showed estrus response 2 to 4 days after CIDR removal. Furthermore, Rasby *et al*. (1998) reported that 80.0% of beef heifers treated with CIDR for seven days exhibited estrus 1 to 3 days after CIDR removal. In addition, Flores *et al*. (2006) found that 56.0% of cows synchronized using CIDR-PGF2α exhibited estrus during the first 3 days of the breeding season.

In the present study, the effect of timed AI on pregnancy rate after estrus synchronization with CIDR, showed that there were no significant differences (P>0.05) among the three treatment groups. However, there was a trend towards increased pregnancy rates from 26.6% (G1) to 24.1% (G2) and 36.6% (G3). These results showed better pregnancy rates after timed AI than in comparison to a previous study where it was reported that the percentage of cows that became pregnant after timed AI at 30 h was only 7.7% (Son *et al.*, 2007).

Timing of insemination is important as it can affect pregnancy rate which is correlated with estrus, ovulation and rates of fertilization (Maquivar *et al.*, 2007). In the present study, high estrus response in G1 was also observed after CIDR removal in all cows. Thus, we can infer that this was not an appropriate time for insemination. Delay in insemination time in G3 after CIDR removal appears to increase pregnancy rate which is probably because of better synchrony with ovulation time. Rajamahendran *et al* (1989) reported that the time ovulation pluriparous and biparous cows were 24 and 30 h, respectively, from the onset of standing estrus.

The increase in conception rate using timed AI at G3 after CIDR removal is still not optimal. The lower rate in this study may have resulted from lactation status in which all cows used in this study were about 50-60 days postpartum. In addition to that, nutrition and management are also very important for conception rate to be successful as suggested by González *et al*. (1988) who observed that suckling and nutritional administration are the main causes of low reproductive efficiency and are also attributed to long calving to conception interval and reduced fertility. Molina *et al*. (2003) reported other factors that affect the percentage of pregnancy rate in cows such as lactational anestrus and erratic reactivation of ovarian activity. In addition, Son *et al*. (2007) reported that lower conception rate resulted from various factors related to lactation status, postpartum interval, and herd nutrition and management. It is therefore concluded that estrus response showed at (D 9) had significant difference from (D 10). Furthermore, timed AI in postpartum beef cattle after removal of a CIDR device resulted in similar pregnancy rate for all time.
